# The Relation of the Non-Protein Nitrogen to the Urea Nitrogen of the Blood

**Published:** 1917-05

**Authors:** 


### The Relation of the Non-Protein Nitrogen to the Urea Nitrogen of the Blood.

This study attempts to interpret the significance of the percentage of the urea nitrogen to the total non-protein nitrogen in the blood in a series of 165 cases. It was found that the percentage of urea nitrogen exhibited a tendency to increase, whether the total non-protein nitrogen were high or low, in all the diseases considered. Cases with acute renal conditions show a high percentage of the total non-protein nitrogen as urea nitrogen of the blood, which returns to normal as convalescence occurs. Individual patients, whose clinical condition does not vary appreciably, exhibit a constant percentage of urea nitrogen, whether the total non-protein nitrogen persists regardless of whether nitrogen is being retained or lost by the body.

The conclusion is drawn that the body usually metabolizes protein in such a manner that approximately 80 per cent of the nitrogen set free in the blood is in the form of urea. The selective action of the kidney maintains the urea nitrogen at a level of 50 per cent or less of the total non-protein nitrogen of the blood. An impairment of renal function, even of very slight degree, may result in an increase of the percentage of urea nitrogen.

From the clinical point of view, figures for urea nitrogen are preferable to those for total non-protein nitrogen because:

(a) The method for urea nitrogen of the blood is simpler.

(b) The methods for urea nitrogen are perfected so that they yield constant results, which are comparable to those of other observers, while this is not true of the various means of determining the total non-protein nitrogen of the blood.—From the *Medical Clinic* of the Johns Hopkins Hospital.

---

School Teacher (to anxious parent): "Your son is bright, intelligent, and getting along in everything but handwriting."

Parent: "That is all right; his writing doesn't matter; I am going to make a doctor of him."—*Medical Reporter.*

